# Effect of Tulathromycin on Colonization Resistance, Antimicrobial Resistance, and Virulence of Human Gut Microbiota in Chemostats

**DOI:** 10.3389/fmicb.2016.00477

**Published:** 2016-04-08

**Authors:** Haihong Hao, Shengxi Zhou, Guyue Cheng, Menghong Dai, Xu Wang, Zhenli Liu, Yulian Wang, Zonghui Yuan

**Affiliations:** ^1^National Reference Laboratory of Veterinary Drug Residues and MAO Key Laboratory for Detection of Veterinary Drug Residues, Huazhong Agricultural UniversityWuhan, China; ^2^MOA Laboratory for Risk Assessment of Quality and Safety of Livestock and Poultry Products, Huazhong Agricultural UniversityWuhan, China

**Keywords:** tulathromycin, gut microbiota, colonization resistance, antimicrobial resistance, chemostat

## Abstract

To evaluate microbiological safety of tulathromycin on human intestinal bacteria, tulathromycin (0, 0.1, 1, 10, and 100 μg/mL) was added into Chemostats. Before and after drug exposure, we monitored (1) population, SCFA products, antimicrobial resistance, and colonization resistance of gut microbiota, and (2) the antimicrobial resistance genes, transferability, virulent genes, pathogenicity of *Enterococus faecalis*. Results showed that low level of tulathromycin did not exhibit microbiological hazard on resistance selection and colonization resistance. However, high level of tulathromycin (10 and 100 μg/mL) may disturb colonization resistance of human gut microbiota and select antimicrobial resistant *E. faecalis*. Most of the selected resistant *E. faecalis* carried resistant gene of *ermB*, transferable element of Tn*1545* and three virulence genes (*esp, cylA*, and *ace*). One of them (*E. faecalis* 143) was confirmed to have higher horizontal transfer risk and higher pathogenicity. The calculated no observable adverse effect concentration (NOAEC) and microbiological acceptable daily intake (mADI) in our study was 1 μg/mL and 14.66 μg/kg.bw/day, respectively.

## Introduction

Tulathromycin is the first and only member of the triamilide sub-class of macrolide. Tulathromycin is used therapeutically in treatment of respiratory disease in swine and cattle at a single dose of 2.5 mg/kg.bw. It has been registered in more than 30 countries across America, Europe, Oceania, and Asia and played important role in veterinary medicine (FDA, 2004). This agent is characterized by rapid absorption from the injection site, extensive distribution to tissue, and slow elimination. The withdrawal period for cattle and swine is 22 and 5 days, respectively. This drug is excreted primarily unchanged (90%) in feces (2/3) and in urine (1/3), suggesting that it may reach to human colon and remain its antimicrobial activity (Benchaoui et al., 2004; EMEA, 2004; Nowakowski et al., 2004).

The residue of tulathromycin in animal food may have unintended microbiological effects on human gut microbiota. The possible harmful effects may be: (1) shifts in bacterial counts and biochemistry, (2) destruction of colonization resistance, (3) emergence of antimicrobial resistant bacteria (Cerniglia and Kotarski, 1999; Nutsch et al., 2005). Additionally, antibiotic resistant bacteria may have enhanced fitness or high virulence (Mundy et al., 2000). Human intestinal strains may also serve as reservoirs for antimicrobial resistant determinations and mediates the gene transfer (Salyers et al., 2004).

Based on *in vitro* antibiotic susceptibility tests, microbiological ADIs recommended by Food and Drug Administration (FDA), European Medicine Agency (EMA), and Australian Pesticides and Veterinary Medicines Authority (APVMA) were 50, 10.99, and 5 μg/kg.bw/d, respectively. The large difference in these mADI may due to the disadvantage and limitation of short-term *in vitro* antibiotic susceptibility tests in evaluation of long-term effects of tulathromycin residues posed on human intestinal bacteria and in analyze of impact of tulathromycin on colonization resistance and emergence of antimicrobial resistance of human colonic microbiota (Cerniglia and Kotarski, 2005). Therefore, microbiological safety assessment measures of tulathromycin residues is largely inadequate. It is largely unknown that what is the effect of long-term exposure to tulathromycin on colonization resistance of human intestinal microbiota and on antimicrobial resistance development, virulence change, and gene transfer in some specific intestinal bacteria.

In the present article, the chemostat model of human colonic gut, which is an approach recommended by VICH (VICH GL-36) and some previous studies (Carman and Woodburn, 2001; Carman et al., 2004, 2005), is used to evaluate microbiological safety of tulathromycin on human intestinal microbiota.

## Materials and methods

### Chemicals

The tulathromycin (TUL), erythromycin (ERY), and lincomycin (LIN) were dissolved in methanol and then diluted with deionized water. The ciprofloxacin (CIP), tetracycline (TET), and rifampicin (RIF) were dissolved in deionized water. Tulathromycin was purchased from Liu He animal Pharmaceutical Co., Ltd. (Qingdao, China). The erythromycin, lincomycin, ciprofloxacin, tetracycline, and rifampicin were purchased from SIGMA.

### Bacteria strains

The *E. coli* ATCC 25922, *Enterococcus faecalis* ATCC29212, and *Micrococcus luteus* ATCC 9341 were purchased from American Type Culture Collection (ATCC). The *E. coli* ATCC 25922 and *E. faecalis* ATCC29212 were used as quality controls for species identification and minimum inhibitory concentration (MIC) determination. The *M. luteus* ATCC 9341 were used as an indicator for determination of the concentration of tulathromycin. *E. faecalis* JH2-2 (Rifampicin MIC > 50 μg/mL) and *E. coli* NK5449 (Rifampicin MIC > 100 μg/mL), purchased from Belgian coordinated collections of microorganisms and Institute of microbiology in Chinese academy of sciences, respectively, were used as receptors for conjugation test. The *Salmonella* Typhimurium SI3 was a ciprofloxacin resistant strain which was selected in our previous study (Sun et al., 2011). This strain was used as the challenge strain for evaluating the colonization resistance of chemostat.

### Preparation of fresh fecal samples and strain isolation

Fresh fecal samples from six adult volunteers who had no history of antibiotic usage and gastrointestinal disturbance within the preceding 3 months were prepared following previous method (Carman and Woodburn, 2001; Hao et al., 2013). The use of human fecal samples was approved by the ethical Committee of Huazhong Agricultural University (approval number hzauch 2013-002).

### Strain isolation and MIC determination from fresh fecal samples

The four predominant intestinal bacteria (*E. coli, Enterococcus, Bifidobacterium*, and Bacteroides *fragilis*) were isolated from the fresh fecal samples of six adult volunteers by selective agars (Eosin methylene blue agar, bile esculin azide agar, BBL media, and bacteroides bile esculin agar). Ten isolates of each species were obtained from fresh fecal sample of each volunteer. After species identification by classic biochemical tests and ABI 3130 system (Applied Biosystem, USA), 32 isolated strains of each species were subjected to MIC determination by agar dilution method provide by CLSI (document M7-A5 for aerobic bacterial and document M11-A5 for anaerobic bacterial). The *E. coli* ATCC25922 and *E. faecalis* ATCC 29212 were used as quality control for MIC determination. The minimum concentration to inhibit 50% of isolates (MIC_50_) and the minimum concentration to inhibit 90% of isolates (MIC_90_) was calculated based on the MIC distribution and estimated by probability analysis using SPSS statistical package (Kays and Graff, 2002; Xu et al., 2013).

### Designation of tulathromycin dosage in preliminary test

The preliminary test for selection of tulathromycin concentration was carried out following the method in previous study (Hao et al., 2013). Briefly, different concentrations of tulathromycin (0, 0.5, 10, 20, 40, 60, 80, 100 μg/mL) were mixed with 50 mL prepared fecal samples at the final concentration of 20% (w/v). The activity of tulathromycin on populations of four different bacteria (*E. coli, Enterococcus, Bifidobacterium*, and *B. fragilis*) were determined by bacterial colony count using selective agars. The highest concentration of tulathromycin was selected for further experiments as it was able to significantly change the population of four predominant intestinal bacteria.

The low to intermediate concentration of tulathromycin (0.1, 1, and 10 μg/mL) were designed based on the acceptable daily intake (ADI) recommended by FDA (50 μg/kg.bw/d), EMA (10.99 μg/kg.bw/d), and APVMA (5 μg/kg.bw/d). In those designed concentration, 1 μg/mL tulathromycin was the mean MIC_50_-value to the most sensitive *Bifidobacterium* strains. One group was drug free methanol control (0 μg/mL tulathromycin).

### Establishment of chemostats and the time schedule

The establishment of chemostat models and the time schedule were carried out following the method in previous studies (Carman and Woodburn, 2001; Hao et al., 2013). Briefly, 50 mL prepared fecal suspension at the final concentration of 20% (w/v) was inoculated into each culture vessel. After 7 h incubation, culture medium was pumped into and out of the culture vessel at a uniform rate of 35 mL/h to maintain 500 mL of culture medium.

After 7 days running, the chemostats reached to a steady state. From 7^th^ to 13^th^ day, the chemostats were kept on running on steady state without administration of tulathromycin(Chen et al., 2011, 2014). From 14^th^ to 20^th^ day, five designed concentrations of tulathromycin (0, 0.1, 1, 10, and 100 μg/mL) were, respectively, infused into the culture medium. During 20^th^–22^nd^ day, the tulathromycin was withdrawn from chemostat models and 1 mL of 6 × 10^8^ CFU/mL (2X McFarland) *Salmonella* Typhimurium SI3 were daily inoculated into each chemostat. Then the chemostats continued to run for another 7 days (to 29^th^ day) without challenge of *Salmonella* Typhimurium (Moffatt, 2007).

### Monitoring bacterial counts in the chemostats

During day 7–20, samples were daily taken from each chemostat. The changes of four predominant bacteria (*E. coli, Enterococcus, B. fragilis*, and *Bifidobacterium*) were measured by viable cell counting (Hao et al., 2013, 2015).

### Monitoring short chain fatty acids (SCFAs) in the chemostats

During day 7–20, samples were daily taken from each chemostat. The concentration of three primary SCFAs (acetic acid, butryric acid, and propionic acid) were monitored using a developed gas chromatographic (GC) method (Hao et al., 2013, 2015).

### Evaluation of colonization resistance in the chemostats

From day 20^th^ to 29^th^, samples were taken from each chemostat to monitor the population of *Salmonella* Typhimurium SI3 using bismuth sulfite (BS) agar containing 4 μg/mL ciprofloxacin(Sun et al., 2011; Hao et al., 2013, 2015). The colonization resistance was disrupted if *Salmonella* Typhimurium SI3 was successfully colonized into chemostat models.

### Monitoring resistance rate in the chemostats

During day 8–20, samples were daily taken from each chemostat. The change of resistance rate was also monitored following the method in previous study (Hao et al., 2013, 2015). The resistance rates of *E. coli* and *Enterococcus* were equal to the number of resistant colonies on the selective agars containing 4-fold MIC_90_ of tulathromycin divided by the number of total colonies on the selective agars without tulathromycin. The 4-fold MIC_90_ was used for selection of tulathromycin resistance on basis of VICH GL-36 document and some previous reports of resistance selection (Allen and Bierman, 2009; Kadlec et al., 2011).

### Determination of resistance pheno- and geno-type in the selected *E. faecalis*

During day 8–20, a total of 70 Enterococcus isolates were random selected from samples in the chemostat containing 100 μg/mL tulathromycin. After species identification by biochemical tests and PCR amplification, 64 isolates were identified as *E. faecalis.* Among these *E. faecalis*, 34 isolates were obtained before the administration of tulathromycin, while 30 isolates were selected after the treatment with tulathromycin. The susceptibility of the 64 *E. faecalis* to macrolide, lincosamides, and tetracycline were determined by agar dilution method.

The genes involved in macrolide-lincosamides-streptogramins (MLS) resistance with a methylation mechanism were determined by PCR amplification of known *erm* genes using specific primers for *ermA, ermB*, and *ermC* (see Table 1). The genes involved in antibiotic efflux systems were determined using specific primers for the *mefA/E* gene (see Table 1). The PCR procedure was followed the method in previous published paper (Portillo et al., 2000).

**Table 1:** **PCR primers and products for detection of macrolides resistance gene and transposons and virulence determinants in *E. faecalis***.

**Primers**	**5′–3′**	**Target fragment (bp)**	**GeneBank no.**
*ermA*	F: GTTCAAGAACAATCAATACAGAG	421	FN668375
	R: GGATCAGGAAAAGGACATTTTAC		
*ermB*	F: GAAAAGGTACTCAACCAAATA	639	NC_014498
	R: AGTAACGGTACTTAAATTGTTTAC		
*ermC*	F: AATCGGCTCAGGAAAAGG	534	NC_014498
	R: ATCGTCAATTCCTGCATG		
*mef (A/E)*	F: AGTATCATTAATCACTAGTGC	346	NC_018641
	R: TTCTTCTGGTACTAAAAGTGG		
Tn *1545*	F: CTTAGAAGCAAACTTAAGAGTGTGT	382	NC_013644
	R: GGTTGAGTA CCTTTTCATTCGTTAA		
Tn *917*	F: ATCTGACGGTGACATCTCTC	652	NC_017312
	R: GGTTGAGTACCTTTTCATTCGTTAA		
*esp*	F: TTGCTAATGCTAGTCCACGACC	932	AF034779
	R: GCGTCAACACTTGCATTGCCGA		
*gelE*	F: ACCCCGTATCATTGGTTT	405	M37185
	R: ACGCATTGCTTTTCCATC		
*cylA*	F: GACTCGGGGATTGATAGGC	688	AD1CLYL
	R: GCTGCTAAAGCTGCGCTTAC		
*ace*	F: GGAATGACCGAGAACGATGGC	616	AF159247
	R: GCTTGATGTTGGCCTGCTTCCG		
*asal*	F: CCAGCCAACTATGGCGGAATC	529	SFPASA1
	R: CCTGTCGCAAGATCGACTGTA		

### Detect transfer risk of the selected macrolide resistant *E. faecalis*

The macrolide-resistant isolates containing *ermB* gene were selected to determine the presence of transposons Tn*1545* and Tn*917* using the primers (see Table 1) and method established in previous study (Okitsu et al., 2005). Filter mating method was used to investigate the conjugative transfer of the selected macrolide resistant *E. faecalis*. A representative macrolide-resistant isolate containing *ermB* and composite transposon was selected as donor strain. The recipients were two rifampicin-resistant strains, *E. faecalis* JH2-2 (Rif MIC > 50 μg/mL) and *E. coli* NK5449 (Rif MIC > 100 μg/mL). The donor and recipient strains were mixed into the conjugation system at the ratio of 1:9 and incubated on the serum agar for 48 h at 37°C. The number of donor, recipient and transconjugants were counted by agar plate containing rifampicin and/or erythromycin.

### Determination of virulence in the selected *E. faecalis*

The presence of virulence factors, including the surface protein (*esp*), haemolysin activator (*cylA*), gelatinase (*gelE*), collagen binding protein (*ace*), and aggregation substances (*asal*), were determined by PCR in the 64 *E. faecalis* isolates obtained in the pre- and post-treatment of tulathromycin in the chemostat. PCR primers used in this study were listed in Table 1. The annealing temperatures of five virulence factors were 65°C for *esp*, 51°C for *gelE*, 61°C for *CylA*, 61°C for *ace* and 63°C for *asal* gene, respectively.

In order to compare the changes of pathogenicity of *E. faecalis*, two representative *E. faecalis* isolates (*E. faecalis* 143 and *E. faecalis* 174) were subjected to determine the median lethal dose (LD_50_). Briefly, the 72 SPF Kunming mice with body weight of 25–28 g were randomly divided into nine groups with half males and half females. The groups were, respectively, challenged with *E. faecalis* isolate by intraperitoneal injection of 0.5 mL diluted concentration of bacteria suspension (10^7^–10^10^ CFU/mouse). The dead mice were carefully dissected in a bacteria-free operating environment. The LD_50_ was calculated by improved Karber method. All experimental procedures were performed according to the guidelines of the committee on the use and care of the laboratory animals in Hubei province China. The study was approved by the Animal Care Center, Hubei Science and Technology Agency in China. All the animals were monitored throughout the study for any sign of adverse effect.

### Statistical analysis

All experiments were performed in triplicate. The statistical analysis was performed following the previous study (Hao et al., 2013, 2015). Briefly, a “mean pre-treatment” level and 95% prediction interval (PI95) for each parameter was set up based on the data obtained from each culture vessel during steady state (day 7–13) and used as reference boundaries for assessing the effect of tulathromycin on each parameter in the drug exposure period (day 14–20).

### NOAEC and mADI calculation

The NOAEC was the no observable adverse effect concentration in this study. The microbiological acceptable daily intake (mADI) for tulathromycin was calculated following the guideline of VICH GL-36. The formula used for mADI calculation was that mADI = NOAEC × daily fecal bolus/(fraction of oral dose available for micro-organisms × weight of human).

## Results

### MIC_90_ of the strains isolated from fresh fecal samples

The 32 strains of each species (*E. coli, Enterococcus, Bifidobacterium*, and *B. fragilis*) were isolated from fresh fecal samples of six volunteers. The MIC for 90% of isolates (MIC_90_) of tulathromycin for *E. coli, Enterococcus, Bifidobacterium*, and *B. fragilis* were 5.53, 7.87, 1.35, and 2.30 μg/mL, respectively. After dilution, the methanol used for dissolving tulathromycin did not exert some antimicrobial effect.

### The designed four tulathromycin concentration

From the preliminary test, 100 μg/mL was the highest concentration of tulathromycin which could significantly change the population of four predominant intestinal bacteria. Based on the ADI-values recommended by FDA, EMA, and APVMA, the lower concentration of tulathromycin were designed as 0.1, 1, and 10 μg/mL.

### Effect of tulathromycin on bacterial counts

**Figure 1:**
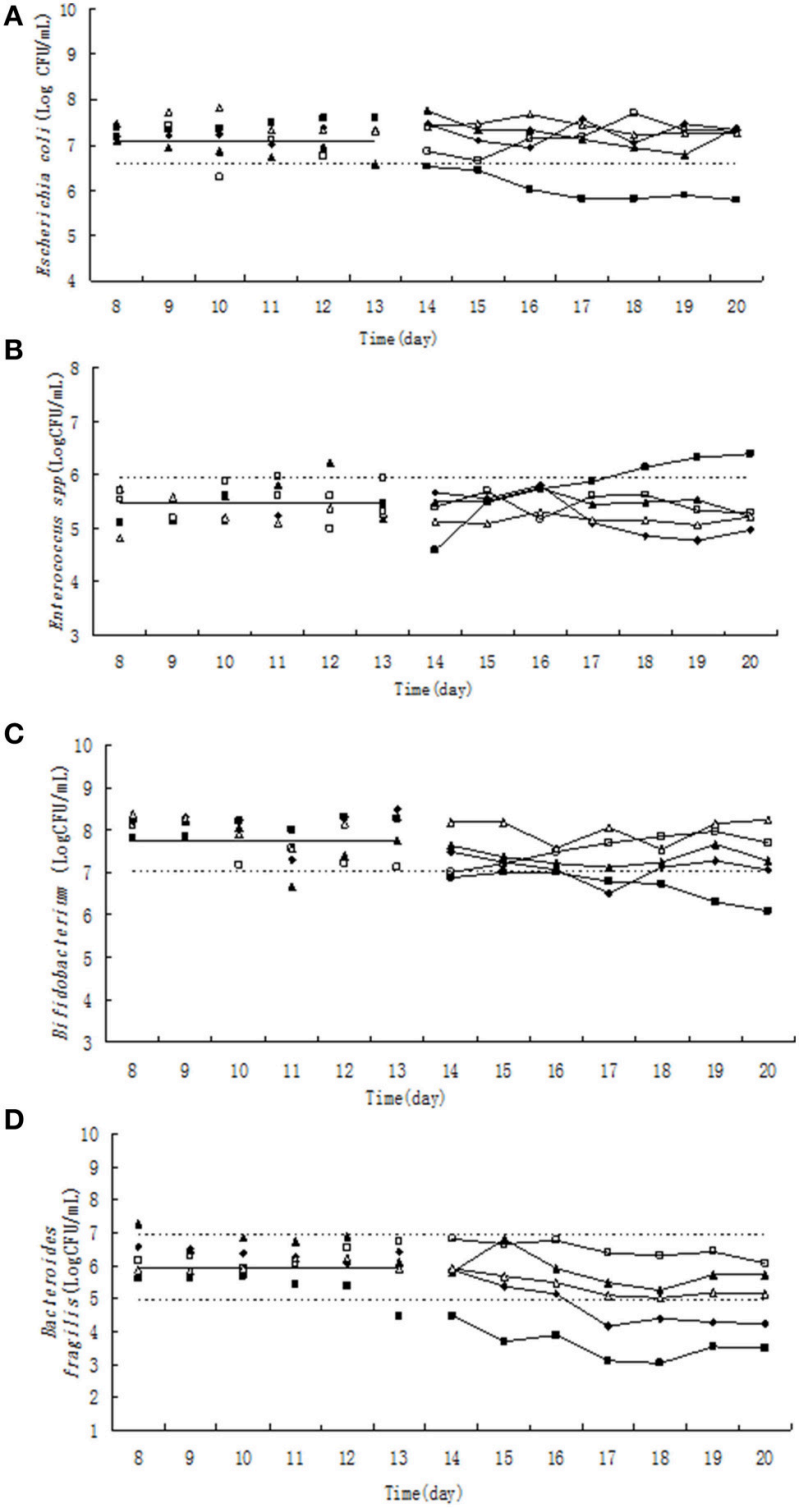
**Effect of tulathromycin on *Escherichia coli* (A), *Enterococcus* (B), *Bifidobacterium* (C), *Bacteroides fragilis* (D) in chemostat model**. □, 0 μg/ mL; Δ, 0.1 μg/ mL; ▴, 1 μg/mL; ♦, 10 μg/mL; ■, 100 μg/mL; —————, pre-treatment mean; - - - - - - - -, 95% predicated interval.

The population of *E. coli* was kept between 3.667 × 10^6^ and 5.482 × 10^6^ CFU/mL in steady state based on statistical data among five chemostats (Figure 1A). Three days after administration of 100 μg/mL tulathromycin, the population of *E. coli* was decreased to 6.556 × 10^5^ CFU/mL which was ~6-fold lower than 95% lower confidence limit. However, the three low concentration of tulathromycin (0.1, 1, and 10 μg/mL) did not significantly change the number of *E. coli*.

Pre-treatment of tulathromycin (day 8–13), the *Enterococcus* counts was in the range of 9.526 × 10^4^–8.674 × 10^5^ CFU/mL (Figure 1B). Under exposure to 10 μg/mL tulathromycin, *Enterococcus* count was decreased ~1.6-fold in the 5^th^ day of post-treatment. On the contrary, the total number of *Enterococcus* was increased almost 3-fold from 18^th^ day after administration of 100 μg/mL tulathromycin.

The number of *Bifidobacterium* varied from 1.105 × 10^7^ to 2.70 × 10^8^ CFU/mL (Figure 1C) during day 8–13. The administration of 100 μg/mL tulathromycin significantly decreased the number of *Bifidobacterium* in the chemostat. It was shown that *Bifidobacterium* count was decreased ~10-fold lower than 95% lower confidence limit.

Significant and dose-dependent effects of tulathromycin on *B. fragilis* counts were observed in all tested concentration of tulathromycin. After treatment by 10 and 100 μg/mL tulathromycin, *B. fragilis* count was decreased 6- and 90-fold lower than 95% lower confidence limit, respectively (Figure 1D).

### Effect of tulathromycin on short chain fatty acid (SCFA)

**Figure 2:**
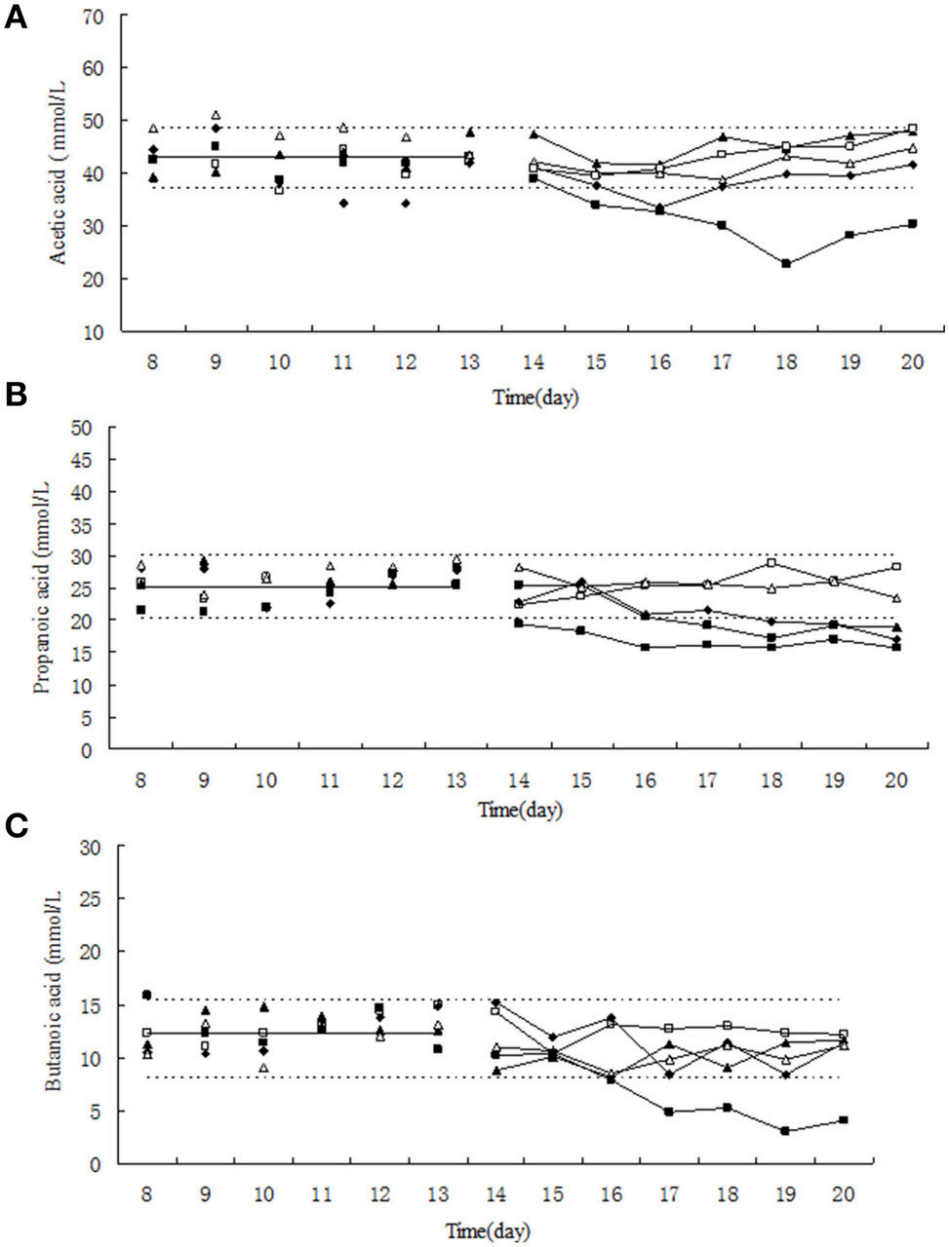
**Effect of tulathromycin on acetic acid (A), propionic acid (B), butyric acid (C) in chemotat models**. □, 0 μg/mL; Δ, 0.1 μg/mL; ▴, 1 μg/mL; ♦, 10 μg/mL; ■, 100 μg/mL; —————, pre-treatment mean; - - - - - - - -, 95% predicated interval.

The relative low concentration of tulathromycin (0, 0.1, and 1 μg/mL) did not significantly change the molar concentration of acetic acid, propionc acid and butyric acid (Figure 2). Under exposure to 10 μg/mL tulathromycin, the concentration of acetic acid and propionc acid was slightly decreased. Upon the administration of 100 μg/mL tulathromycin, the concentration of acetic acid, propionc acid, and butyric acid were decreased ~1.3, 1.3, and 2.68-fold lower than 95% lower confidence limit, respectively. Among the three dominant SCFAs, the concentration of propionc acid was changed remarkably under exposure of tulathromycin and present significant dose-dependent effect of tulathromycin.

### Effect of tulathromycin on colonization resistance

**Figure 3:**
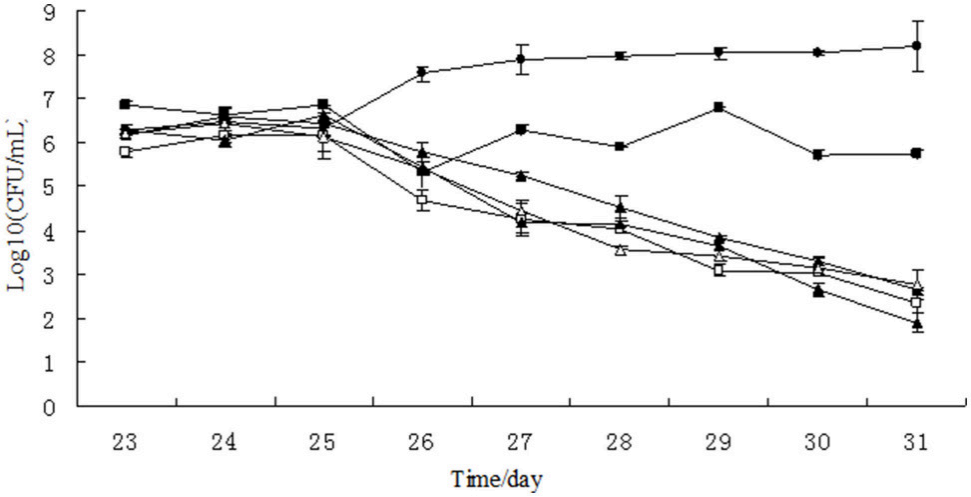
**Growth curves of *Salmonella* Typhimurium in chemostat models**. □, 0 μg/mL; Δ, 0.1 μg/mL; ▴, 1 μg/mL; ♦, 10 μg/mL; ■, 100 μg/ mL; ∙, growth control.

In the single growth control group, the growth of *Salmonella* Typhimurium was stable at the level of 3.75 × 10^7^–1.54 × 10^8^ CFU/mL, indicating that *Salmonella* Typhimurium grew well in the chemostat model containing no drug and gut microbiome (Figure 3). However, the colony number of *Salmonella* Typhimurium tapered to 10^2^ CFU/mL on day 31 in the chemostat containing stable gut microbiome, suggesting that the gut microbiome may establish colonization resistance to exogenous pathogens. After treatment of lower concentration of tulathromycin (10, 1, and 0.1 μg/mL), the number of *Salmonella* Typhimurium was also decreased to the level of 10^2^ CFU/mL. In contrast, the colonization number of *Salmonella* Typhimurium was in the range of 2.08 × 10^5^–7.15 × 10^6^ CFU/mL in the chemostat administrated by highest concentration of tulathromycin (100 μg/mL), suggesting that the high concentration of drug may disturb the colonization resistance of gut microbiome and induce the proliferation of pathogen (Figure 3).

### Effect of tulathromycin on resistant rate

**Figure 4:**
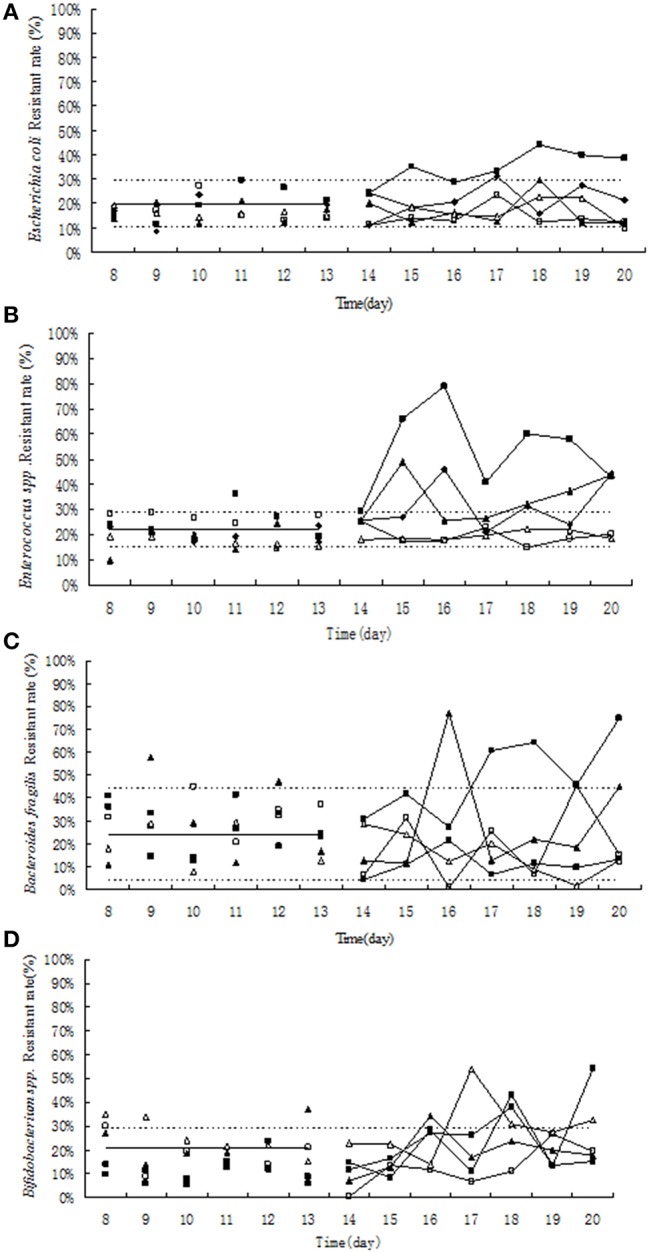
**Effect of tulathromycin on resistance in *Escherichia coli* (A), *Enterococcus* (B), Bifidobacterium (C), *Bacteroides fragilis* (D) in chemostat model**. □, 0 μg/mL; Δ, 0.1 μg/mL; ▴, 1 μg/mL; ♦, 10 μg/ mL; ■, 100 μg/ mL; —————, pre-treatment mean; - - - - - - - -, 95%predicated interval.

Before the treatment of tulathromycin, 10–30% of tulathromycin resistance was observed in *Escherichia coli* and in *Enterococcus* strains (Figures 4A,B). Under exposure to 10 μg/mL tulathromycin, the percentage of resistant *Enterococcus* was slight increased (Figure 4B). After treatment with 100 μg/mL tulathromycin, the resistant rates in *E. coli* and *Enterococcus* were obviously higher than control group, especially in *Enterococcus*, it reach up to 80% (Figures 4A,B). After administration of different concentration of tulathromycin, regular changes were not observed in *Bifidobacterium* and *Bacteroides fragilis* (Figures 4C,D).

### Resistant phenotype and genotype of the selected *E. faecalis*

After species confirmation, 34 and 30 *E. faecalis* isolates were selected from chemostats pre- and post-treatment, respectively. The MIC_50_ and MIC_90_ of these 64 *E. faecalis* strains to erythromycin, tulathromycin, lincomycin, and tetracycline were showed in Table 2. The 34 *E. faecalis* strains isolated from chemostats before administration of tulathromycin exhibited high susceptibility to erythromycin, tulathromycin and tetracycline (MIC ≤ 4 μg/mL), and low resistance to lincomycin (MIC = 16, 32 μg/mL). However, after treatment with tulathromycin, 30 *E. faecalis* isolates (except for the strains of 141, 142, 144, and 164) showed high-level resistance to the four tested antibiotics (MIC ≥ 4 μg/mL). The MIC_50_ of erythromycin, tulathromycin, lincomycin, and tetracycline to *E. faecalis* were considerably increased for 88.9, 49.8, 5.1, and 45.4-fold, respectively.

**Table 2:** **Comparison of MICs of separated *E. faecalis* strains, before and after treated with four drugs**.

**Drug**	**Pre- or post-treatment**	**Strains (*N*)**	**MICs (μg/mL)**											**MIC_50_ (μg/mL)**	**MIC_90_ (μg/mL)**
			**256**	**128**	**64**	**32**	**16**	**8**	**4**	**2**	**1**	**0.5**	**0.25**		
ERY	Pre-	34						1	2	26	4		1	2.610	5.211
ERY	Post-	30	17	9						3	1			231.698	—
TLU	Pre-	34							31	2			1	4.904	10.995
TLU	Post-	30	21	4					3	2				244.149	—
LIN	Pre-	34				27	6						1	41.706	221.77
LIN	Post-	30	21	1	2	6								213.721	669.13
TET	Pre-	34								33			1	2.552	4.437
TET	Post-	30		22	2					5		1		115.681	—

MIC_50_ = minimum concentration to inhibit 50% of isolates; MIC_90_ = minimum concentration to inhibit 90% of isolates. MIC_90_- and MIC_50_-values are estimated values by probability analysis by SPSS (Kays and Graff, 2002; Xu et al., 2013); “—” datas not given; white areas was pre-treatment group; gray areas was post-treatment group; ERY, erythromycin; TLU, tulathromycin; LIN, lincomycin tetracycline.

About 88.46% of the tulathromycin resistant *E. faecalis* isolated from chemostats harbored macrolide resistance associated gene of *ermB* (Table 3). However, the tested macrolide resistance associated genes (*ermABC* and *mefA/E*) were free in the tulathromycin resistant *E. faecalis* strains (Table 3).

**Table 3:** **The relationship of MIC, resistant gene and virulence gene in *E. faecalis* isolated during drug administration**.

**Isolates no**.	**MICs**				**Resistant gene**		**Virulence determinants**			
	**ERY**	**TLU**	**LIN**	**TET**	***ermB***	**Tn1545**	***esp***	***cylA***	***gelE***	***ace***
141	2	4	32	2	–	–	–	–	+	+
142	2	4	32	2	–	–	–	–	+	+
143	128	256	256	128	+	+	+	+	–	+
144	2	4	32	2	–	–	–	–	+	+
145	256	256	32	2	–	+	–	–	+	+
151	128	256	256	128	+	–	+	+	–	+
152	128	128	256	128	+	+	+	+	–	+
154	1	2	32	2	–	–	–	–	+	+
155	128	128	32	0.5	–	–	–	–	+	+
161	256	256	256	128	+	+	+	+	–	+
162	256	256	256	128	+	+	+	+	–	+
164	256	256	256	128	+	+	+	+	–	+
165	256	256	256	128	+	+	+	+	–	+
171	256	256	256	128	+	+	+	+	–	+
172	128	256	64	64	+	–	–	–	–	–
174	256	256	64	64	–	+	–	–	–	–
181	128	256	256	128	+	+	+	+	–	+
182	256	256	256	128	+	+	+	+	–	+
183	256	256	256	128	+	+	+	+	–	+
185	256	256	256	128	+	+	–	–	–	–
191	128	256	256	128	+	+	+	+	–	+
192	128	128	256	128	+	+	+	+	–	+
193	256	256	256	128	+	+	+	+	–	+
194	256	256	256	128	+	+	+	+	–	+
195	256	256	256	128	+	+	+	+	–	+
201	128	128	128	128	+	+	+	+	–	+
202	256	256	256	128	+	+	+	+	–	+
203	256	256	256	128	+	+	+	+	–	–
204	256	256	256	128	+	+	+	+	–	+
205	256	256	256	128	+	+	+	+	–	+

The isolate was numbered by isolated day and strain number. For example, isolate 143 was the third isolate obtained in 14^th^ day. Gray shades used to distinct the positive and negative of special virulence factor.

### Transfer risk of macrolide resistant *E. faecalis*

All of *ermB* containing *E. faecalis* strains also harbored composite transposon of Tn*1545* (Table 3). When the *E. faecalis* 143 strain containing both *ermB* and Tn*1545* was selected as donor strain and subjected to conjugation transfer test, the result showed that macrolide resistant determinants could transfer to *E. faecalis* JH2-2 and *E. coli* NK5449 at the frequencies of 10^−6^–10^−7^ (Table 4).

**Table 4:** **The frequencies of conjugation transfer between donor and host strains**.

**Receptor strains**	**Times**	**Donor strain (CFU/mL)**	**Host strain (CFU/mL)**	**Transconjugant (CFU/mL)**	**Transfer frequency**
*E. faecalis JH2-2*	1	1.21 × 10^9^	2.62 × 10^8^	2.04 × 10^3^	7.79 × 10^−6^
	2	4.30 × 10^9^	6.75 × 10^8^	9.40 × 10^2^	1.39 × 10^−6^
	3	3.72 × 10^9^	4.35 × 10^8^	2.60 × 10^3^	5.98 × 10^−6^
*E. coli NK5449*	1	8.42 × 10^8^	6.82 × 10^8^	1.90 × 10^2^	2.79 × 10^−7^
	2	1.25 × 10^9^	3.71 × 10^8^	2.20 × 10^2^	5.93 × 10^−7^
	3	1.13 × 10^9^	1.65 × 10^8^	6.60 × 10^2^	4.00 × 10^−6^

### Virulence of the selected *E. faecalis*

**Figure 5:**
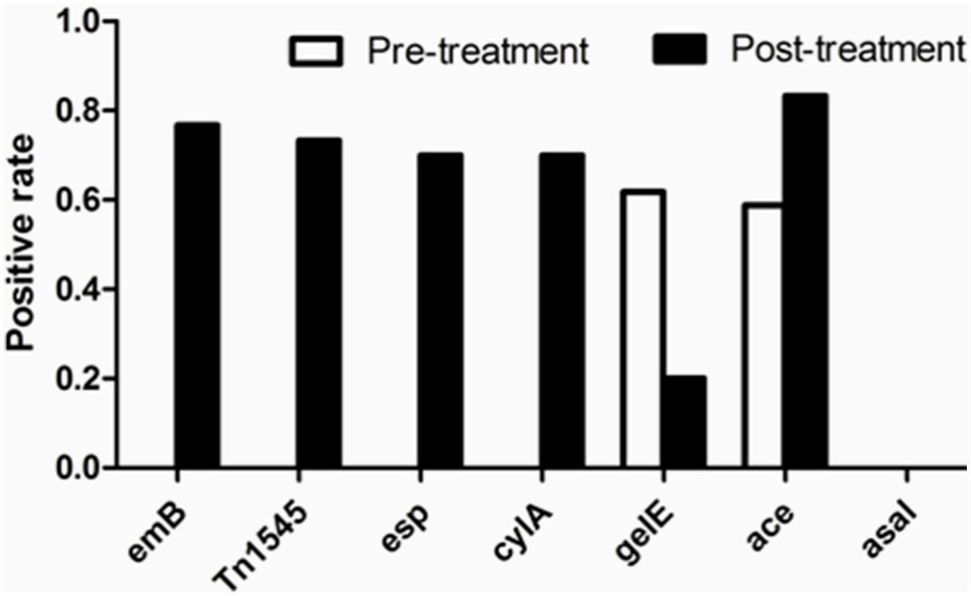
**Comparison of positive rate of resistant determinants and virulence genes of *E. faecalis* before and after experiment with drug**.

In the 34 *E. faecalis* stains selected in the pre-treatment, positive ratios of *esp, gelE, clyA, ace*, and *asal* were 0, 61.76, 0, 58.8% and 0, respectively. However, these changed into 70, 20, 70, 83.3% and 0, respectively, in the 30 *E. faecalis* stains selected in the post-treatment (Figure 5). The positive rates of surface protein (*esp*) and hemolysin activator (*cylA*) were significantly increased (0–70%). To the contrary, the carriage ratios of gelatinase (*gelE*) had a downside (61.76–20%). Aggregation substances (*asal*) were not found in all of the 64 strains *E. faecalis*. As shown in Table 3, most of the antimicrobial resistance in *E. faecalis* selected after treating with tulathromycin carried resistance gene of *ermB*, transferable element of Tn*1545* and three virulence gens (*esp, cylA*, and *ace*).

After infection with *E. faecalis* 143 (harboring *ermB*, Tn*1545, esp, cylA*, and *ace*), mice died within 8 h which was earlier than the death of mice infected with *E. faecalis* 174 (free of *ermB, esp, cylA*, and *ace*). The LD_50_ of the two *E. faecalis* strains was calculated by improved Karber method. The detailed results were shown in Table 5. The LD_50_ *E. faecalis* 143 (5.970 × 10^9^ μg/mL) was three-times higher than that of *E. faecalis* 174(1.603 × 10^9^ μg/mL), suggesting that *E. faecalis* 143 strain harboring virulence factors (*esp, cylA*, and *ace*) was more virulent than the strain without these two virulence genes.

**Table 5:** **Comparing the LD_50_ of *E. faecalis* 143 and 174 in the mice experiment**.

***E. faecalis* no**.	**Inoculated dose (CFU/mouse)**	**Total mice number**	**Dead mice number during experiment**				**lgLD_50_**	**LD_50_ (CFU/mL)**
			**0–8 h**	**8–24 h**	**24–48 h**	**>48 h**		
14-3	1.60 × 10^10^	8	7	0	0	0	9.205	1.603 × 10^9^
	1.60 × 10^9^	8	3	1	1	0		
	1.60 × 10^8^	8	0	0	0	0		
	1.60 × 10^7^	8	0	0	0	0		
	0	8	0	0	0	0		
17-4	1.06 × 10^10^	8	2	1	1	0	9.776	5.970 × 10^9^
	1.06 × 10^9^	8	0	0	1	0		
	1.06 × 10^8^	8	0	0	0	0		
	1.06 × 10^7^	8	0	0	0	0		
	0	8	0	0	0	0		

### NOAEC and mADI calculation

Based on our results, the NOAEC was set up as 1 μg/mL. The daily fecal bolus was known as 220 mL. The fraction of oral dose available for microorganisms in the intestinal tract was 0.25 which can be split into two factors of 0.5 each. One factor of 0.5 was based on results for reduced availability of the substance due to interaction with fecal matter (EMEA, 2004) and the other factor of 0.5 was based on the impact of acidic colonic pH on tulathromycin availability for gut bacteria(USFDA, 2004). The weight of human was 60 kg/person. Therefore, our established mADI was 14.66 μg/kg.bw/d.

## Discussion

This study is the first of its kind to assess the effect of tulathromycin on human microbiota using the chemostat model. In addition, this study concentrated on the antimicrobial resistance development and virulence change of *E. faecalis* isolates in chemostats under long term exposure to a range of tulathromycin concentrations.

The present study showed that the intermediate concentration (10 μg/mL) slightly change the population of *Enterococci* and *Bacteroides fragilis*, while the highest concentration (100 μg/mL) inhibited the growth of three type of bacteria except for *Enterococcus*. This result was similar with our previous results that the numbers of some intestinal bacteria (e.g., *B. fragilis*) underwent significant changes during exposure to tilmicosin (Hao et al., 2015). As one of the most predominant bacteria in the intestinal microbiota, *B. fragilis* may be a most sensitive indicator of gut microbiome under exposure to macrolide drugs including erythromycin, tilmicosin, and tulathromycin (Carman et al., 2005; Merck sharp and D. Corp., 2011; Hao et al., 2015).

The present study and our previous investigation suggested that in the chemostat model high concentration of macrolide drugs (tilmicosin and tulathromycin) could significantly reduce the population of *B. fragilis* and subsequently resulted into the decline of the three main SCFAs especially propionic acid (Hao et al., 2015). The related change of propionic acid with *B. fragilis* population that we found was also observed in human flora associated (HFA) mice model (MacNeil, 2005).

Similar to previous studies, the colonization resistance of human intestinal microbiota in the chemostat was disrupted under 7 days exposure to high concentration of antibiotics (Carman et al., 2004; Hao et al., 2013). Coincidently, in our chemostat model, there were significant alterations in the populations of *E. coli, B. fragilis, Enterococcus*, and *Bifidobacterium* which appeared to be indicator of intestinal microbiota and contributed a lot to the colonization resistance (Corpet, 1993; Nuding et al., 2013). As previously reported, 10^5^ CFU/mL of *E. coli* had a complete inhibitory effect on the growth of *Salmonella* (Carman et al., 2004). However, in our previous result, the treatment of tilmicosin did change the colonization resistance (Hao et al., 2015). This difference suggested that disruption of colonization resistance was largely depended on antimicrobial agents and their concentrations (Carman et al., 2004; Ferreira et al., 2011).

Similar to previous results, resistance rate in *E. coli* did not change in the chemostat and HFA rodent models treated by tilmicosin (EMEA, 1997; Cerniglia and Kotarski, 2005; Hao et al., 2015). This may be due to the inherent resistance to macrolide drugs in *E. coli* (Phuc Nguyen et al., 2009). However, the resistance rate of *Enterococcus, Bifidobacterium*, and *B. fragilis* was significantly increased under the selective pressure of 100 and 10 μg/mL tulathromycin in our study. Consistently, the subtherapeutic and therapeutic administration of tulathromycin also significantly increased the proportion of erythromycin resistant enterococci in beef cattle (Zaheer et al., 2013).

Our study showed that most of the isolated tulathromycin resistant enterococcicontained *ermB*. The *ermB* gene in different gram-positive bacteria has been well-documented (Schmitz et al., 2000; Perreten et al., 2005; Littauer et al., 2006; Tremblay et al., 2011; Zmantar et al., 2011). The occurrence of macrolide resistance mediated by *ermB* was also found in enterococci originating from swine and cattle due to the subtherapeutic use of tylosin and tulathromycin (Jackson et al., 2004; Chen et al., 2008; Zaheer et al., 2013).

Tn*1545* was found in almost of the high-level macrolides resistant *Enterococcus* harboring *ermB* gene. Our conjugation test also showed that the *Enterococcus* isolate containing Tn*1545* and *ermB* can transfer its resistance to *E. faecalis* JH2-2 and *E. coli* NK5449. The transferability of *ermB* located in transposon may play an important role on the increase of resistance rate in *Enterococcus, Bifidobacterium*, and *B. fragilis* (Okitsu et al., 2005; Ciric et al., 2013).

Both the previous study and our study found that *gelE* gene was one of the predominant virulence genes in *E. faecalis* (Di Rosa et al., 2006). A positive correlation between macrolide resistance and *gelE* virulence gene was observed in an epidemiological investigation (Zou et al., 2011; Lins et al., 2013). High level expression of *gelE* has also been observed in multi-drug resistant Enterococci and in macrolide resistant *E. feacalis* containing *ermB* gene (Arciola et al., 2008; Hao et al., 2015). However, the *E. feacalis* containing both *ermB* gene and gelatinase (*gelE*) did not occur in our study.

Most of *E. faecalis* harboring both virulence factors (*esp* and *cylA*) and resistance gene of *ermB* were selected after treatment with tulathromycin, however, no *E. faecalis* isolates containing these two virulence genes was found before tulathromycin treatment. Coincidently, recent studies also found the existence of a large pool of potentially virulent and multidrug resistant *E. faecalis* in diseased farm animals (Seputiene et al., 2012). The presence and expression of some virulence determinants (e.g., *esp* and *cylA*) may enhance the colonization and invasion of *E. faecalis* to the epithelial cell of host (Trieu-Cuot et al., 1990; Littauer et al., 2006; Heikens et al., 2009; Johanson et al., 2012; Cafini et al., 2015; Kafil and Mobarez, 2015). The acute toxicity test with SPF mice indicated that *E. faecalis* harboring the macrolide resistant gene of *ermB* and two virulence genes (*esp* and *cylA*) had higher toxicity and pathogenicity.

Our established mADI (14.6 μg/kg.bw/d) was slightly higher than the mADI (10.97 μg/kg.bw/d) recommended by EMA-CVMP and the mADI (5 μg/kg.bw/d) recommended APVMA. Recently, EMA-CVMP revised the mADI of tulathromycin to 55 μg/kg.bw/d (EMA/CVMP, 2015). As a new approved drug, more work of safety assessment needs to be done to establish the final mADI of tulathromycin.

## Executive summary

**Table:** 

**Dose dependent effect on colonization resistance**
10 μg/mL tulathromycin would significantly decrease the population but increase the resistance rate of *Enterococcus* and *Bacteroides fragilis*.
100 μg/mL tulathromycin significantly decreased population of *Escherichia coli, Bifidobacterium* and *B. fragilis* and SCFAs.
The colonization resistance was disturbed by higher concentration of tulathromycin.
**Antimicrobial resistance inducement and risk of resistant factors**
10 μg/mL tulathromycin would significantly increase the resistance rate of *Enterococcus* and *Bacteroides fragilis.*
100 μg/mL tulathromycin largely increased resistant rate of *Enterococcus* and selected multidrug resistant *E. faecalis*.
The selected resistant *E. faecalis* always carried *ermB* gene. It was located in transposons Tn*1545* and had ability of horizontal gene transfer (HGT).
**The virulence change under drug exposure**
The *E. faecalis* harboring resistance gene of *ermB* gene and virulence gene of *esp* and *cylA* was only selected after administration of high concentration of tulathromycin.
The *E. faecalis* harboring *esp* and *cylA* had higher pathogenicity.
**Microbiological acceptable daily intake (mADI) of tulathromycin**
The mADI was calculated as 14.66 μg/kg bw/day based on our study.

## Conclusions

In conclusion, the established NOAEC and mADI in our study was 1 μg/mL and 14.66 μg/kg.bw/day, respectively. The low concentration of tulathromycin had no significant microbiological hazard with regard to colonization resistance and antimicrobial resistance. However, the long-term exposure to high concentration of tulathromycin (100 μg/mL) may damage the colonization resistance of human gut microbiota and induce the development of antimicrobial resistance in *Enterococcus*. The *E. feacalis* containing both transferable resistance determinant (*ermB* in Tn*1545*) and virulence genes (*esp* and *cylA*) were selected after administration of high level tulathromycin. More work need to be done to systematically assessment the safety of tulathromycin on human public health.

## Author contributions

Experiment designation: HH, SZ, YW, and ZY; Experiment implement: SZ, HH, and ZL; Data analysis: SZ, HH, XW, YW, and ZY; Manuscript writing: SZ and HH; Manuscript modification: HH, GC, MD, YW, and ZY.

## Funding

This work was supported by Grants from National Basic Research program of China (2013CB127200), project supported by the morning program of Wuhan in China (2015070404010191), Fundamental Research Funds for the Central Universities (2662015PY035), National Natural Science Foundation of China (31101856 and 31272614), and National Program for Risk Assessment of Quality and Safety of Livestock and Poultry Products (GJFP2016008).

### Conflict of interest statement

The authors declare that the research was conducted in the absence of any commercial or financial relationships that could be construed as a potential conflict of interest.
